# First IncHI2 Plasmid Carrying *mcr-9.1*, *bla*_VIM-1_, and Double Copies of *bla*_KPC-3_ in a Multidrug-Resistant Escherichia coli Human Isolate

**DOI:** 10.1128/mSphere.00302-21

**Published:** 2021-05-28

**Authors:** Serena Simoni, Marina Mingoia, Andrea Brenciani, Maria Carelli, Maria M. Lleò, Giovanni Malerba, Carla Vignaroli

**Affiliations:** a Department of Life and Environmental Sciences, Polytechnic University of Marche, Ancona, Italy; b Department of Biomedical Sciences and Public Health, Polytechnic University of Marche, Ancona, Italy; c Department of Diagnostics and Public Health, University of Verona, Verona, Italy; d Department of Neurosciences, Biomedicine and Movement Sciences, University of Verona, Verona, Italy; Escola Paulista de Medicina/Universidade Federal de São Paulo

**Keywords:** colistin, carbapenems, multidrug resistance, IncHI2 plasmid, *Escherichia coli*

## Abstract

We report a novel IncHI2 plasmid coharboring *bla*_VIM-1_, two copies of *bla*_KPC-3_, and *mcr-9.1* resistance genes in a human Escherichia coli isolate of the new serogroup O188. The *bla*_VIM-1_ gene was included in a class 1 integron, *mcr-9.1* in a cassette bracketed by IS*903* and ΔIS1R, and *bla*_KPC-3_ in two copies within a new composite Tn*4401*-like transposon. The emergence of carbapenem and colistin resistance genes in a single plasmid is of great concern for upcoming clinical therapies.

## OBSERVATION

Carbapenems are considered antibiotics of choice against multidrug-resistant and extended-spectrum β-lactamase-producing strains, but the global increase of carbapenemase-producing *Enterobacteriaceae* (CPE) are compromising their use in therapy (1). Carbapenemases are frequently encoded by genes located on transferable elements and isolates of Escherichia coli, Klebsiella, and Enterobacter spp., carrying multiple carbapenemase-encoding genes on plasmids of different incompatibility (Inc) groups, have been reported (1). Colistin is often the last-line antibiotic against serious CPE infections; however, CPE strains with mobilized colistin resistance (*mcr*) determinants are emerging worldwide (2), further limiting the current therapeutic options.

In this study, we report the first human multidrug-resistant E. coli isolate (Ec3) coharboring *bla*_VIM-1_ and two copies of *bla*_KPC-3_ and *mcr-9.1* genes on the same IncHI2 plasmid. Ec3 was previously described as a highly resistant strain to imipenem, meropenem, and ertapenem (MICs of >128 μg/ml) but susceptible to colistin (MIC of 0.12 μg/ml) and tigecycline (MIC of 0.12 μg/ml), belonging to the sequence type ST1266 and PCR positive to *bla*_VIM-1_ and *bla*_KPC-2_ (3). S1 nuclease pulsed-field gel electrophoresis (S1-PFGE) and following hybridization assays showed that *bla*_VIM-1_ and *bla*_KPC-2_ were located on a plasmid of ∼250 kb. The unusual coharboring of two carbapenemase genes on the same plasmid prompted us to fully investigate the strain by whole-genome sequencing.

Genomic analysis performed using both the Illumina (Technological Platform Center of the University of Verona, Italy) and Oxford Nanopore DNA sequencing platforms (MicrobesNG, Birmingham, UK) revealed a genome consisting of 5.282,753 bp with a 50.5% GC content and the presence of a 249,437-bp plasmid with a 48% GC content. Sequencing data showed Ec3 strain belonged to E phylogroup and to O188:H34 serotype. Interestingly, the O188 serogroup, recently recognized in E. coli, shows a new O-antigen polysaccharide almost identical to Shigella boydii type 16 (4), suggesting the potential pathogenicity of Ec3 strain. In addition, some virulence genes (*pap*, *afaD*, and *chuA*) involved in adhesion and iron acquisition in diarrheagenic and uropathogen E. coli strains (5), were found by VirulenceFinder analysis on the Ec3 chromosome.

ResFinder analysis confirmed the strain carried multiple genes mediating resistance to beta-lactams (*ampC*, *bla*_ACC-1_, *bla*_OXA-1_, *bla*_KPC-3_, and *bla*_VIM-1_), aminoglycosides [*aadA1*, *aph(3*′′*)-Ib*, and *aph(6)-Id*], fluoroquinolones [*aac(6*′*)-Ib3*], sulfonamides (*sul1* and *sul2*), trimethoprim (*dfrA24*), chloramphenicol (*catA1*), tetracycline (*tetB*), and also to colistin (*mcr-9.1*), although the strain was phenotypically susceptible to polymyxins. Remarkably, the *mcr-9.1*, *bla*_KPC-3_, and *bla*_VIM-1_ genes were located on the same plasmid (pEC3).

**FIG 1:**
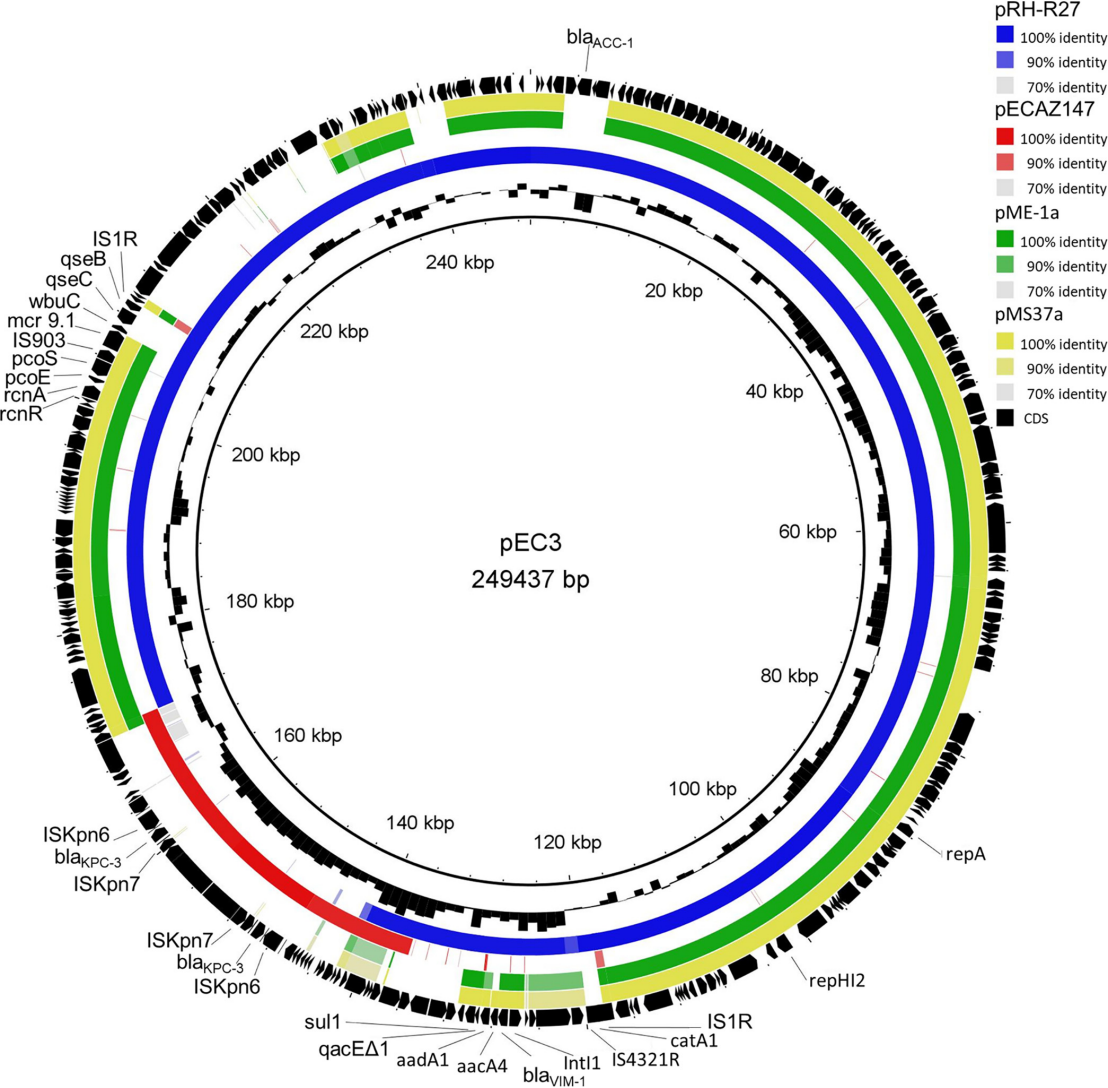
Genetic characterization of the IncHI2 plasmid pEC3. Circular map of the pEC3 plasmid coharboring *mcr-9.1*, *bla*_VIM-1_, and *bla*_KPC-3_ in comparison with similar reported plasmids using BRIG software. The plasmids included in the analysis were as follows: (inner to outer circles) pRH-R27 (GenBank ID LN555650), pECAZ147_KPC (CP018992), pME-1a (CP041734), and pMS37a (CP053191). Black arrows indicate the positions and orientations of genes; some resistance and relevant genes described in this study are shown.

The pEC3 (GenBank accession no. MW509820) carried the IncHI2 replicon and belonged to the ST1 (6), showing the highest coverage (88%) and nucleotide identity (100%) with the VIM-1 and MCR-9 encoding plasmid pRH-R27 (GenBank accession no. LN555650) of Salmonella enterica isolated from a livestock farm in Germany (7). By BLASTN, pEC3 revealed similarity also to previously described IncHI2 plasmids carrying *bla*_VIM_ and *mcr-9* genes, like pME-1a (72% coverage and 99.97% identity, GenBank accession no. CP041734 [8]) and pMS37a (66% coverage and 99.97% identity, accession no. CP053191 [9]) from human or food Enterobacter hormaechei isolates, respectively (Fig. 1).

All beta-lactamase genes, except for *ampC* and *bla*_OXA-1_, were located on pEC3. The *bla*_ACC-1_ gene, an AmpC-type beta-lactamase originated from Hafnia alvei, was associated with an IS*Ecp1* element as commonly reported for *Enterobacteriaceae* (10). The genetic context and sequence of *bla*_ACC-1_ were the same as in pRH-R27.

**FIG 2:**
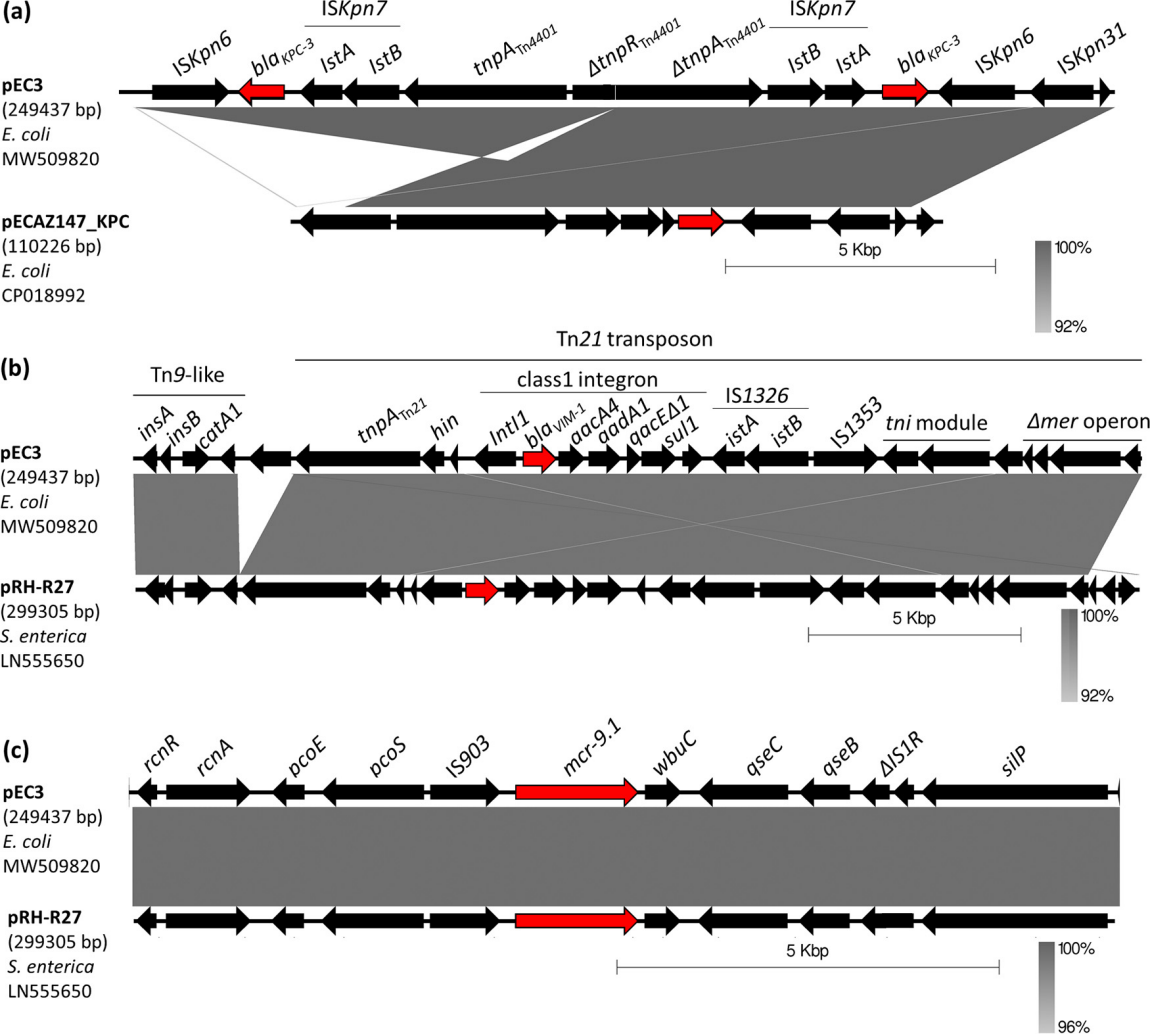
(a to c) Linear comparison of the *bla*_KPC-3_, *bla*_VIM-1_, and *mcr-9.1* contexts of pEC3 with the corresponding regions of highly similar plasmids pECAZ147_KPC and pRH-R27. Gray shading indicates regions of shared homology (ranging from 92 or 96% to 100%). The three resistance genes under study are shown by red arrows.

The *bla*_KPC-3_ gene, improperly reported as *bla*_KPC-2_ in the previous study (3), was bracketed by the IS*Kpn7* (upstream) and the IS*Kpn6* (downstream) within a Tn*4401a* transposon as first described by Naas et al. (11). However, Tn*4401a* was disrupted by the insertion into the *tnpR* gene of a further copy of the same truncated transposon in opposite direction (Fig. 2a). This arrangement was probably due to a recombination event between the two copies of Tn*4401a* transposon. The result was the loss of a 3,126-bp fragment and the formation of a composite transposon (of 16,688 bp) delimited by two identical IS (IS*Kpn6*) and containing two *bla*_KPC-3_ genes. The highest nucleotide similarity was observed with a portion of the IncF plasmid pECAZ147_KPC (accession no. CP018992) from a human E. coli. Adjacent copies of Tn*4401*-like transposons on the same plasmid have been reported in Klebsiella pneumoniae (12, 13), but their combination in a single transposable element has not yet been reported.

The *bla*_VIM-1_ gene was included in a class 1 integron almost identical to that carried by the plasmid pRH-R27 of S. enterica. The integron variable region contained the *bla*_VIM-1_, *aacA4*, and *aadA1* gene cassettes. As in pRH-R27, the integron was in a Tn*21* transposon, also included in a Tn*9* homolog harboring the *catA1* gene for chloramphenicol resistance. However, in pEC3, a truncated *mer* operon downstream of the *tni* module of integron was observed (Fig. 2b).

The core structure of *mcr-9.1* cassette “*rcnR-rcnA-pcoE-pcoS*-IS*903*-*mcr-9.1*-*wbuC*” was identical to that described in other IncHI2 plasmids in different *Enterobacteriaceae* (14) and very similar to that of pRH-R27 (100% coverage and 99.96% identity). The regulatory genes (*qseC* and *qseB*), followed by IS*1R*, were detected downstream of the *wbuC* gene (Fig. 2c). Nevertheless, induction experiments, using subinhibitory concentrations of colistin (0.03 to 0.06 μg/ml), followed by quantitative real-time PCR (RT-qPCR) assays performed as described by Kieffer et al. (15), caused no increase of *mcr-9* gene expression and no higher MIC to colistin. The role of *qseC-qseB* on *mcr-9* induction may differ in isolates with different genetic backgrounds as suggested by Tyson et al. (16), but other genes may also be involved in the regulation of *mcr-9* expression (8, 14).

Since IncHI transfer rate is temperature dependent (17), conjugal experiments were performed at 37°C and 25°C, but they were both unsuccessful, consistent with deletions in one of the transfer regions. In particular, the *dsbC* and *traI* genes encoding a thioredoxin-like protein and relaxase, respectively, both involved in IncHI2 plasmid transfer, were not found in pEC3, as in a nonconjugative plasmid variant (pRH-R178) of pRH-R27 (7).

In conclusion, we describe in the new O188 E. coli serogroup, a novel IncHI2 plasmid coharboring *mcr-9.1*, *bla*_VIM-1_, and *bla*_KPC-3_. It likely originated by recombination with elements frequently associated with IncF plasmids and although nonconjugative, demonstrates that the ongoing spread of *mcr-9* and carbapenemase genes is caused by their association with genetic contexts able to move in different plasmids and bacteria.
